# The development of a dietary nutrient density educational tool and the investigation of its acceptance by Chinese residents from Henan province

**DOI:** 10.1186/s12889-024-20222-4

**Published:** 2024-10-04

**Authors:** Junya Zhai, Xu Zhang, Pipasha Khatun, Saiqi Wang, Minghua Cong, Rui Liang, Fangfang Yao, Huan Liu, Jin Qin, Lijun Guo, Yongxia Kong, Hongbo Wu, Baihui Ma

**Affiliations:** 1 Department of Clinical Nutrition, Affiliated Cancer Hospital of Zhengzhou University, Henan Cancer Hospital, Zhengzhou, China; 2 Department of Nutrition and Food Hygiene, College of Public Health, Zhengzhou University, Zhengzhou, Henan China; 3 Department of Comprehensive Oncology, National Clinical Research Center for Cancer/Cancer Hospital, National Cancer Center, Chinese Academy of Medical Sciences and Peking Union Medical College, Beijing, China; 4 Department of Clinical Nutrition, The First Affiliated Hospital of Zhengzhou University, Zhengzhou, China; 5 Department of Clinical Nutrition, The Second Affiliated Hospital of Zhengzhou University, Zhengzhou, China; 6 Department of Public Health, Zhengzhou Shuqing Medical College, Zhengzhou, China; 7 Health Management Center, Affiliated Cancer Hospital of Zhengzhou University, Henan Cancer Hospital, Zhengzhou, China

## Abstract

**Objectives:**

Helping residents select nutrient-dense foods is a strategy to improve their diet quality. However, communication based on the nutrient-dense foods as a positive attribute has not been widely used in nutritional education. This study aimed to develop an educational tool based on the picture and guidance of “Chinese food guide pagoda (2022) “, extend it with the concept of nutrient density, and investigate its acceptance by Chinese residents from Henan province.

**Methods:**

Three examples (one-day diet with high, medium, and low nutrient-rich food (NRF) 9.2 score, an indicator for evaluating dietary nutrient density) were designed for developing a dietary nutrient density educational tool. A self-designed questionnaire was conducted to investigate the acceptance of the “dietary nutrient density educational tool” among college students from Henan province on the basis of the theory of planned behavior.

**Results:**

Among the three one-day diets used in the tool, with the decrease in the NRF9.2 score, the energy intake increased from 1686 kcal to 2363 kcal, the dietary fat-to-energy ratio increased from 28 to 42%, and the mean adequacy ratio (MAR) decreased from 0.97 to 0.87. A total of 851 college students completed the acceptance questionnaire. The average score of the acceptance was 4.07, with a total score of 5. This study showed that resident’s intention to use the tool was correlated with family residence, perceptual behavior control, and subjective norms. These three factors accounted for 83.5% of the variation in behavior intention.

**Conclusion:**

To encourage residents choosing healthier foods, a dietary nutrient density educational tool was developed to expanding the current instructional tool—the Chinese food guide pagoda (2022). The acceptance questionnaire survey revealed that residents had good acceptance of the tool, and family residence, perceptual behavior control, subjective norms may strongly contribute to their acceptance and the intention to use of the tool.

**Supplementary Information:**

The online version contains supplementary material available at 10.1186/s12889-024-20222-4.

## Introduction

The global burden of non-communicable diseases (NCDs) has increased in the last decades, and China is no exception. From 2002 to 2018, the prevalence of obesity (7.1–16.4%), hypertension (18.8–27.5%) and diabetes (2.6–11.9%) were dramatically increased [1]. The percentage of deaths caused by NCDs was 80.0% in 2002 and 88.5% in 2018 [1]. Much of the burden of NCDs is preventable through the modification of lifestyle behaviors, and increased attention is being focused on identifying and implementing effective preventative health strategies [2]. Nutrition has been identified as a major modifiable determinant of NCDs [3]. The nutrition transition in China, characterized as decreased intake of cereals and vegetables, and increased intake of animal foods with pork dominating, and the consumption of cooking oil and salt was dramatically far above the recommendations [4], substantially impacts the dietary quality and health outcomes of Chinese residents, reflecting discernible shifts in dietary patterns and nutritional profiles [5]. To address this issue, the dietary guidelines, a series of versions published by the Chinese Nutrition Society, guide Chinese residents to eat healthily [6]. Jiajie Zang et al. reported that maintaining a healthy and balanced diet, as reflected in adherence to the Chinese Dietary Guidelines (CDGs), is related to increased overall health among Shanghai residents [7]. Shi-Xiu Zhang et al. confirmed that nutritional education guided by the “Dietary Guidelines for Chinese Residents” had beneficial effects on anthropometric, lipid, adipokine, and inflammatory markers in metabolic syndrome patients in Shanghai [8].

Promoting a healthy diet through education is part of the Healthy China 2030 action plan. The dietary guidelines for Chinese residents are excellent educational documents, that guide dietary intake [9]. The dietary guidelines for Chinese residents (2022) are composed of five food groups and its corresponding quantity including cereals and tubers, vegetable and fruit, lean meat, poultry, aquatic products and egg, soy beans, nuts and milk, salt and cooking oil [10, 6], which also suggests choosing highly nutrient-dense foods as noted in the message of the dietary guidelines for Americans and the USDA MyPyramid from 2005 [11]. Nutrient-dense foods have been described as providing substantial amounts of nutrients for relatively few calories or offering fewer calories than nutrients [12]. Helping consumers select nutrient-dense foods is a strategy to improve their diet quality [13]. The nutrient-rich food ( NRF ) index is a valid tool for assessing the nutrient density of individual foods and assessing the total diet [10, 14, 15]. Diets with high NRF index scores protect against central obesity [16] and a high body mass index (BMI) [10, 17], and are inversely associated with all-cause mortality [18]. However, communication based on the nutrient density of foods as a positive attribute has not been widely used in nutritional education. Amy R Moble designed the NRF educational tool “My5” to demonstrate how the NRF approach offers people a way to take small steps toward meeting daily food and nutrient needs within calorie limits [19]. Karen Glanz et al. reported that a consumer education program increased participants’ use of the NRF approach and improved diet quality [20]. On the basis of the above research, this study focused on developing an educational tool that incorporates the quantity of the recommended food by the Chinese food guide pagoda (2022) and extended it with the concept of nutrient density, which will be applied to individual foods, help consumers build meals, and track choices across days, weeks, or longer to ultimately create a healthier diet. Moreover, this study took Chinese college students from Henan province as an example to investigate the acceptance of the tools by residents.

## Subjects and methods

### Developing a “dietary nutrient density educational tool”

#### The concept of developing a “dietary nutrient density educational tool”

To create a method of choosing reasonable foods on the basis of nutrition density that would make it simple for residents to choose and enjoy healthier meals by receiving the most nutrition from their calories, Drewnowski A. established the NRF index in 2003. The NRF index successfully ranks foods on the basis of their nutritional value and can be applied to individual foods, meals, menus, and even the one-day diet [21]. The NRF9.2 index, a family of NRF indices, taking nine beneficial nutrients and two nutrients to limit into account, has been validated against the mean adequacy ratio (MAR), which is an independent measure of dietary quality among Chinese adults [10]. Therefore, according to the NRF9.2 score of each food from five food groups and the quantity of recommendation from Chinese food guide pagoda (2022), one-day diets with low, medium, and high NRF9.2 score were developed on the basis of 2000 kcal. A “dietary nutrient density educational tool” was developed on the basis of the Chinese food guide pagoda (2022) and extended with the concept of nutrient density.

#### Developing dietary one-day diet with different levels of nutritional quality

Three one-day diets were designed on the basis of the Chinese food guide pagoda and a nutrition calculator with the same quantity of each recommended food based on 2000 kcal but with different NRF9.2 score. The reference nutrients intakes for computing the NRF 9.2 index based on 2000 kcal according to Chinese Dietary Reference Intakes 2013 [22] were presented in Table 1. The quantity of each food category recommended by Chinese food guide pagoda (2022) on the basis of 2000 kcal were given (Table 2). The one-day diet with high, medium, and low NRF9.2 scores were designed through the food replaced by the same food category with different NRF9.2 scores. The nutrient intake was calculated via the Nutrition Calculator software developed by the medical and health nutrition and management platform according to Chinese food composition table [23] , and the NRF9.2 score was subsequently calculated.

**Table 1:** Chinese dietary reference intakes for calculating the NRF 9.2 index on the basis of 2000 kcal

Nutrients	Average level of Reference
Energy(kcal)	2000
Protein (g)	60
Dietary fiber (g)	25
Vitamin A (µgRE)	750
Vitamin C (mg)	100
Vitamin E (mg α-TE)	14
Calcium (mg)	900
Iron (mg)	15
Potassium (mg)	2000
Magnesium (mg)	325
Saturated fat (g)	22.2
Sodium (mg)	1450

**Table 2:** Balanced dietary patterns and food intake for 2000 kcal energy levels (g per person per day)

Food type	Food quantity (g)	Number of blocks	The intake level in the one-day diet
Cereals	250	5	50 g/ block
---Whole grains and mixed beans	50–150		
Tubers*	75(equivalent to a weight of 15 g cereals)		
Vegetables	450	2	225 g/ block
---Dark color vegetables	225	1	225 g/ block
Fruits	300	2	150 g/ block
Meat and poultry	50	1	50 g/ block
Eggs	50	1	50 g/ block
Aquatic products	50	1	50 g/ block
Milk or yogurt	300	1	300 g/ block
Soybeans	15	0.5	15 g /0.5 block
Nuts	10	0.5	10 g /0.5 block
Cooking oil	25	0.5	25 g/0.5 block
Salt	5	0.5	5 g/ block

*Tubers are fresh weight

#### The specific design of the “dietary nutrient density educational tool”

**Fig. 1:**
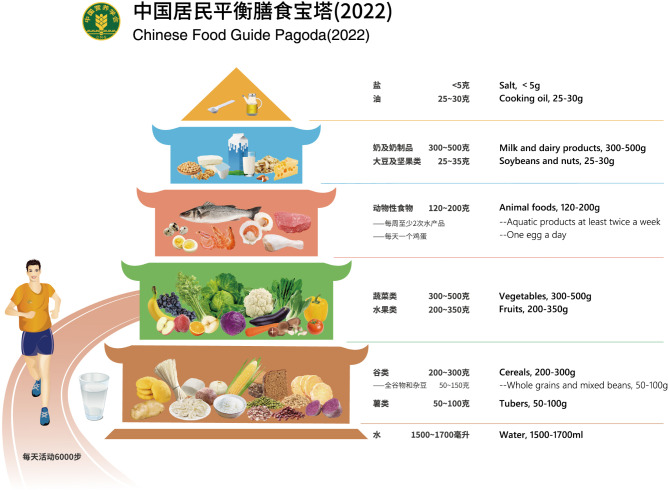
Chinese food guide pagoda (2022) and with its English translation

The “dietary nutrient density educational tool” was developed via a drawing of the Chinese food guide pagoda (2022). The tool conveyed the concept of nutrient density in the following ways. The first was the score of the tool. The foods consumed by Chinese residents from Henan province [10] with different NRF9.2 scores from same food group were divided into four groups based on interquartile range, and assigned 1 to 4 points respectively. 1 point represented the food with the lowest nutritional quality, and 4 points represented the food with the highest nutritional quality. The score for the tool also ranged from 1 to 4 points. The second was the color of the tool. The color of the Chinese food guide pagoda (2022) represented the nutrition density of the food consumed. The more the original color blocks, the greater the nutritional quality. The number and color intensity of the block could be used to communicate the concept of nutrient richness. For example, the recommended intake of cereals on the basis of the 2000 kcal energy level was 250 g, and we designed five blocks at the cereals level, with each block representing 50 g. If NRF9.2 score of the cereals is 4 points, the block will keep its original color (brown); if it is 3 points, three-quarters of the block will keep its actual color, and therefore, one-quarter of the block will become a lighter color. If the score is 2 points, half of the block preserves its actual color, and therefore, the other half of the portion will become a lighter color. If the score is 1 point, one-quarter of the portion retains its color, turning the other three-quarters into a lighter hue. As a result, the nutritional quality increased with the number of blocks that retain the tool’s original color but decreased with the number of light-colored containers. The drawing method of the “dietary nutrient density educational tool” was based on the “Chinese food guide pagoda (2022)” according to the NRF9.2 score of each food and using Photoshop technology. A picture of the " Chinese food guide pagoda (2022)” with the English translation was shown in Fig. 1.

#### Setting the energy label

The energy label of each one-day diet was attached at the bottom of the Chinese food guide pagoda (2022). If the energy of the one-day diet is within the preset energy level (2000 kcal), then the color of the energy block is becoming dark. The proportion of the energy block will be filled with a gray color if the one-day diet’s energy surpasses the preset energy value and a white color is drawn if the energy is less than the preset energy value.

### Dietary quality evaluation of different one-day diets of the “dietary nutrient density educational tool”

#### NRF9.2 score

NRF9.2 index, based on 9 beneficial nutrients and 2 nutrients to limit, using the algorithm based on sums and 100 kcal, was the best-predicted model for Chinese adults, and thus it was selected in this study [10]. The daily reference intakes of nutrients were based on the recommended nutrient intake (RNI) or adequate intake (AI) of adults, except for saturated fat which was based on acceptable macro-nutrient distribution ranges [22]. All foods from one-day diet had scored using the NRF9.2 algorithms, followed by the NRF9.2 score per 100 kcal [24].

$${\rm{NRF}}\,{\rm{9}}{\rm{.2}}\,{\rm{/100kcal = }}\left( {{\rm{NR}}\,{\rm{9 - }}\,{\rm{LIM}}\,{\rm{2}}} \right){\rm{ \times 100}}$$

$${\rm{NR9 = \Sigma 1 - 9}}\left( {{\rm{Nutrien}}{{\rm{t}}_{\rm{i}}}{\rm{/NR}}{{\rm{V}}_{\rm{i}}}} \right){\rm{/ED \times 100}}$$

Nutrient_i_= content of nutrient i in 100 kcal edible portion.

NRVi = Nutrient i based on Chinese Dietary Reference Intakes.

ED = Energy density of 100 g edible portion of food.

i = 1–9 ( protein, dietary fibre, vitamin A, C, E, Ca, Fe, Mg, K ).

LIM2 = Σ1–2( L_i_/MNRV_i_ )/ED×100.

L_i_ = nutrients i content of 100 g edible portion of food.

MNRV_i_ = Maximum reference intake of nutrient i.

i = 1–2( saturated fat, Na ).

When the quantity of food consumed was the same, the higher the NRF9.2 score was, the better the diet quality.

#### The ratio of energy supply

The ratios of the energy supplied from protein, fat, and carbohydrate were recommended to be 10–15%, 20–30% and 50–65%, respectively [6].

#### Assessment of nutrient adequacy

Nutrient adequacy was measured for computing MAR. To compute MAR, nutrient adequacy ratio (NAR) was calculated on the basis of DRIs, the nutrients chosen were given in Table 3. The MAR was calculated as described by Madden et al. [25].

**Table 3:** Chinese dietary reference intakes for calculating NAR and MAR on the basis of 2000 kcal

Nutrients	Average level of reference
Energy (kcal)	2000
Protein (g)	60
Vitamin A (µgRE)	750
Vitamin C (mg)	100
Calcium (mg)	900
Iron (mg)	15
Phosphorous (mg)	700
Vitamin B_1_ (mg)	1.3
Vitamin B_2_ (mg)	1.3
Vitamin PP (mgNE)	14
Vitamin E (mg α-TE)	14
Magnesium (mg)	325
Potassium (mg)	2000
Zinc (mg)	10
Selenium (mg)	50
Fiber (g)	25

$$\:\text{N}\text{A}\text{R}=\frac{\text{A}\text{c}\text{t}\text{u}\text{a}\text{l}\:\text{n}\text{u}\text{t}\text{r}\text{i}\text{e}\text{n}\text{t}\:\text{i}\text{n}\text{t}\text{a}\text{k}\text{e}\:\text{o}\text{f}\:\text{a}\:\text{n}\text{u}\text{t}\text{r}\text{i}\text{e}\text{n}\text{t}\:\left(\text{p}\text{e}\text{r}\:\text{d}\text{a}\text{y}\right)}{\text{C}\text{h}\text{i}\text{n}\text{e}\text{s}\text{e}\:\text{d}\text{a}\text{i}\text{l}\text{y}\:\text{r}\text{e}\text{f}\text{e}\text{r}\text{e}\text{n}\text{c}\text{e}\:\text{i}\text{n}\text{t}\text{a}\text{k}\text{e}\text{s}\:\text{o}\text{f}\:\text{t}\text{h}\text{e}\:\text{n}\text{u}\text{t}\text{r}\text{i}\text{e}\text{n}\text{t}}$$

$$\:\text{M}\text{A}\text{R}=\frac{\sum\:\text{N}\text{A}\text{R}\left(\text{e}\text{a}\text{c}\text{h}\:\text{t}\text{r}\text{u}\text{n}\text{c}\text{a}\text{t}\text{e}\text{d}\:\text{a}\text{t}\:1\right)}{\text{N}\text{u}\text{m}\text{b}\text{e}\text{r}\:\text{o}\text{f}\:\text{n}\text{u}\text{t}\text{r}\text{i}\text{e}\text{n}\text{t}\text{s}}$$

### Investigation of the acceptance of the “dietary nutrient density educational tool”

A self-designed questionnaire was used to investigate the acceptance of the “dietary nutrient density educational tool” among the college students from Henan province on the basis of the theory of planned behavior. According to the theory of planned behavior, individuals’ behavior attitudes, subjective norms, and perceptual behavior control jointly determine their behavior intentions and then affect their behavior [26]. The items were evaluated by a Likert scale 5. The subjects selected the one that most conformed to their cognition from the five options of “strongly agree,” “agree,” “generally,” “disagree,” and “strongly disagree,” and assigned them 5 points, 4 points, 3 points, 2 points, or 1 point, respectively.

A total of 92 college students from one university located at Henan province via convenience sampling method completed the questionnaire for testing reliability and validity. In order to test validity, factor analysis was used. Before factor analysis, Bartlett’ spherieity test and Kaiser-Meyer-olkin (KMO) measurement of sampling adequacy were carried out to test the correlation of each question. In this study, the KMO value was 0.833, and the significance probability of the statistical value of Bartlett sphere test was 0.001, indicating that the data was suitable for factor analysis. The standard extracting common factors was based on eigenvalue (greater than 1) and drawing line becomes flat based on the gravel diagram and then four common factors are extracted. The corresponding eigenvalues, variances and cumulative variances were shown in Table 4.

**Table 4:** Eigenvalue and variance contribution rate of each factor in the acceptance questionnaire (*n* = 92)

Common factor	Eigenvalue	Variance(%)	Cumulative variance(%)
1	5.690	35.563	35.563
2	1.563	9.768	45.331
3	1.387	8.668	53.999
4	1.008	6.300	60.299

The questionnaire was extracted four subdomains: behavior attitude (6 items), subjective norm (6 items), perceptual behavior control (4 items), and behavior intention (2 items). Finally, the acceptance questionnaire included 18 items, and showed satisfactory internal consistency in subdomains with a Cronbach’s α ranging between 0.847 and 0.943. The basic information was also investigated.

A cross-sectional study was conducted by convenience sampling from June 2023 to November 2023. The college students aged 18 to 23 years old from three colleges located in Zhengzhou city was recruited for filling in the acceptance questionnaire. In total, 893 individuals were invited, and 851 individuals (male 35.93%) were included for analysis. A total of 42 individuals with missing data on any item of the questionnaire were excluded.

Body weight (nearest 0.1 kg) and height (nearest 0.1 cm) were measured in duplicate by using an ultrasonic weight and height instrument while the participants were barefoot and wearing light clothes only. BMI was calculated in the standard way. Weight (kg) divided by square of height (m), which was classified as underweight (< 18.5 kg/m^2^ ), normal weight (≥ 18.5 and < 23.9 kg/m^2^), overweight (≥ 24 and < 27.9 kg/m^2^) and obese (≥ 28 kg/m^2^) according to the working group on obesity in China (WGOC) [27].

### Quality control

Quality control was carried out from questionnaire design to data analysis. First, the questionnaire used in the investigation was revised after the pilot study and expert discussion. Second, all investigators underwent standardized training before the interview. Finally, all data were inputted by two persons, and logical error detection and review were carried out.

### Statistical analysis

The drawing method of the “dietary nutrient density educational tool” was based on the “Chinese food guide pagoda (2022)” according to the NRF9.2 score of each food and using Photoshop technology. The questionnaire on the acceptance of the “dietary nutrient density educational tool” was designed and tested for reliability and validity. SAS statistical software, version 9.3 (SAS Institute, Cary, NC, USA), was used for all data analysis. A *p* value < 0.05 was considered statistically significant. The tool acceptance was analyzed via multiple linear stepwise regression analysis. If the assumptions of linear regression models failed, the continuous data of behavior intention will be changed to categorical data and then multiple logistic regression analysis will be explored. Potential confounders include age, sex, BMI, family residence, as they perhaps affect consumers using dietary tools [19, 28, 29].

## Results

### Design of the “dietary nutrient density educational tool”

**Fig. 2:**
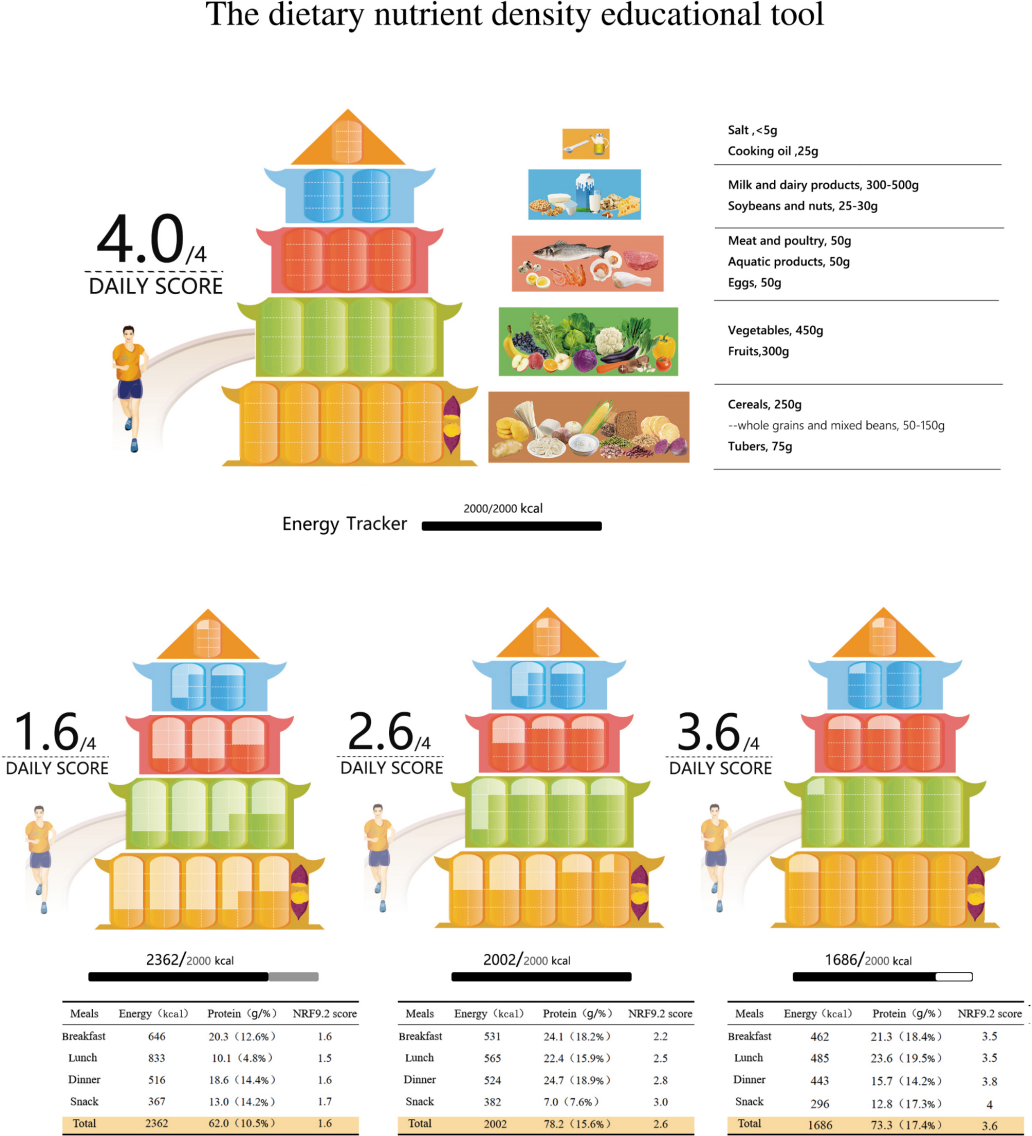
Dietary nutrient density educational tool

The “dietary nutrient density educational tool” was developed by Photoshop technology shown in Fig. 2. It conveys that “choosing reasonable food is the foundation of a healthy diet”. The specific design steps were given in the second part-subjects and methods: “developing a “dietary nutrient density educational tool”.

### Evaluation of the “dietary nutrient density educational tool”

According to one-day diet design principles, one-day diet with high, medium, and low NRF9.2 scores were developed from the recommended food intake of the Chinese food guide pagoda (2022). With the decrease in the score of the one-day diet, the energy intake increased from 1686 kcal to 2363 kcal, and the dietary fat-to-energy ratio increased from 28 to 42% (Table 5).

**Table 5:** Distribution of the energy supply ratio with different NRF9.2 score for one-day diet

	Higher NRF9.2 one-day diet (3.6 score)				Medium NRF9.2 one-day diet(2.6 score)				Lower NRF9.2 one-day diet (1.6 score)		
	Nutrients intake (g)	Energy (kcal)	Energy supply ratio(%)		Nutrients intake (g)	Energy (kcal)	Energy supply ratio(%)		Nutrients intake (g)	Energy (kcal)	Energy supply ratio(%)
Protein	73.33	293.32	17.39		78.22	312.88	15.64		62.01	248.04	10.50
Fat	52.8	475.2	28.19		56.19	505.71	25.26		112.36	1011.24	42.81
Carbohydrate	229.4	917.48	54.42		296	1183.41	59.10		275.63	1102.72	46.69
Total	----	1686	100.00		----	2002	100.00		----	2362	100.00

Among the 15 nutrients, there were 5 nutrients (vitamin B_1_, vitamin E, vitamin pp, calcium and fiber) whose NAR < 1 in the one-day diet with higher NRF9.2 score, and the MAR was 0.97. There were 6 nutrients (vitamin A, vitamin B_1_, vitamin B_2_, vitamin pp, calcium and fiber) whose NAR < 1 in the one-day diet with medium NRF9.2 score (MAR = 0.91). There were 9 nutrients (vitamin A, vitamin B_1_, vitamin B_2_, vitamin C, vitamin pp, magnesium, calcium, zinc and fiber) whose NAR < 1 in the one-day diet with lower NRF9.2 score, and the MAR was 0.87. The results were shown in Table 6.

**Table 6:** Nutrient adequacy rates of different NRF9.2 score one-day diet

One-day diet		Protein	VitA	VitB_1_	VitB_2_	VitC	VitE	VitPP	Potassium	Magnesium	Calcium	Iron	Zinc	Phosphorus	Selenium	Fibre
	RNI^1^	60	750	1.3	1.3	100	14	12	2000	325	900	13\\15	10	700	50	25
With high NRF9.2 score	Actual intake	73.33	1093	1.2	1.31	205	13.16	11.3	2807	475	884	29.46	13.13	1196	53.22	19.27
	NAR^2^	1.00	1.00	0.92	1.00	1.00	0.94	0.94	1.00	1.00	0.98	1.00	1.00	1.00	1.00	0.77
With medium NRF9.2 score	Actual intake	78.22	529	1.02	1.06	160	32.08	11.73	2577	391	772	21.49	11.06	1246	51	13.64
	NAR	1.00	0.71	0.78	0.82	1.00	1.00	0.98	1.00	1.00	0.86	1.00	1.00	1.00	1.00	0.55
With low NRF9.2 score	Actual intake	62.01	406	0.73	1.23	72	35.98	9.93	2528	323	701	19.99	9.66	1084	53.38	17.73
	NAR	1.00	0.54	0.56	0.95	0.72	1.00	0.83	1.00	0.99	0.78	1.00	0.97	1.00	1.00	0.71

Note: 1:RNI: recommended nutrient intake; 2:NAR: nutrient adequacy ratio

### Investigation of the acceptance of the “dietary nutrient density educational tool”

The distribution of general characteristics of the sample and each dimension of the acceptance questionnaire was given in Table 7. It showed that the college students had good acceptance of the tool with average score of 4.07, in which the dimension with the highest score is the attitude, with an average score of 4.12, and the lowest score was perceptual behavior control with an average score of 4.00.

**Table 7:** Distribution of the general characteristics and each dimension of the acceptance questionnaire (*n* = 851)

Variable	Classification	X ± S / *n*(%)^1^
Age		20.26 ± 3.46
Family residence	Urban area	405(47.59%)
	Rural area	446(52.41%)
Sex	Male	306(35.96%)
	Female	545(64.04%)
BMI(kg/m^2^)	≤ 18.5	112(13.16%)
	18.5–24.0	513(60.28%)
	24.0–28.0	130(15.28%)
	≥ 28.0	96(11.28%)
Behavior attitude		4.12 ± 0.71
Subjective norm		4.08 ± 0.75
Perceptual behavior control		4.00 ± 0.77
Behavior intention		4.07 ± 0.80
The average score of acceptance		4.07 ± 0.72

Note:1 X ± S: X = Mean, S = standard deviation

Multiple linear stepwise regression analysis was used to analyze the factors affecting the acceptance of the tool. The results implied that behavior intention was positively correlated with family residence, subjective norms, and perceptual behavior control, in which the role of subjective norms was most substantial, and the three factors explained 83.5% of the variation in behavior intention, but not in behavior attitude (Table 8).

**Table 8:** Analysis of the factors affecting the intention to use healthy food selection tools

Behavior intention^1^	Partial regression coefficient	Standard Error	Standardized partial regression coefficient	t value	*P*	Adjusted *R*^2^	F value	*P*
Family residence	0.048	0.023	0.030	2.049	0.041	0.835	1295.55	0.001
Subjective norm	0.512	0.034	0.476	15.23	0.001			
Perceptual behavior control	0.486	0.033	0.467	14.92	0.001			

Notes: 1. Adjusted age, sex (male/female), BMI(≤ 18.5,18.5–23.9,24.0–28.0,≥28.0), family residence (urban area, rural area)

## Discussion

The “dietary nutrient density educational tool” was developed to help residents make healthier food choices and improve their overall diet quality. The tool was developed on the basis of the picture of “Chinese food guide pagoda (2022)” and extended it with the concept of nutrient density via three examples, demonstrating how the NRF approach offers residents a way to take small steps toward meeting daily food and nutrient needs within calorie limits. The concept conveyed by this tool was to choose nutrient-dense food among same food groups following the Chinese food dietary guideline (2022). In addition, the acceptance of the “dietary nutrient density educational tool” was investigated. The results showed that behavior intention was correlated with family residence, subjective norms, and perceptual behavior control. It suggested that the acceptance of the tool can be increased by residents through the influence of authoritative people around them or by providing sufficient conditions.

Residents are advised to seek nutrient-dense foods to meet nutrient requirements without exceeding daily energy needs, which is a strategy to improve their diet quality [13]. Not limited to individual foods only, the NRF algorithm can be applied to food groups, meals, menus, and total diets, which describes the concept that helps residents make wise decisions on their food choices—both inside and outside the store [21]. The “dietary nutrient density educational tool” exemplifies how the NRF index illustrated the concept of nutrient density. This approach may change the food purchasing patterns of residents, leading to measurable changes in diet quality in the future. We plan to develop an app based on the NRF9.2 score, which gave the NRF9.2 score of each food and can easily help residents calculate the NRF 9.2 score of the meal or even the total diet. If the NRF 9.2 score of one-day diet was unoptimistic during recipes designing, residents can change foods with lower scores by foods with higher scores among the same food groups for improving diet quality. However, this view needs to be spread among the public. Amy R. Mobley et al. developed an educational tool named “My5” based on the NRF index score, which built a balanced diet and extended it with the concept of nutrient density [19]. As Shirley A Gerrior noted that nutrition education which included easy-to-use scientific information about the nutritional quality of foods, such as information provided by nutrient profiling systems, can improve resident’s decision-making about healthy foods [30]. Therefore, this study put the concept of nutrient density into the Chinese food guide pagoda (2022) for permeating the concept of selecting nutrient-dense foods as an important method to build a healthier diet for the residents. With the development of the nutrient density calculation app by our research team on the basis of the NRF9.2 score, the concept of helping residents make healthier food choices can be realized.

This study revealed that the behavioral intention to use a “dietary nutrient density educational tool”  was positively correlated with family residence, subjective norms, and perceptual behavior control, in which the role of subjective norms was most vital, and the three factors can explain 83.5% of the variation in behavior intention. Feifei Huang et al. reported that having higher education, greater income, living in an urban area, and being aware of the Chinese dietary guidelines (2016) recommendations were positively associated with adherence to Chinese dietary guidelines [31]. Yangyang Sun et al. reported that individuals with a lower level of education and rural residents benefit more from increasing their dietary knowledge [32]. Therefore, the acceptance and the willingness to use the educational tools can be increased for the rural residents by providing health education to them. Subjective norms are the pressure of individuals from relatives, friends, colleagues, and authority persons when they implement a specific behavior and whether they are willing to obey these pressures [33]. The people around him or her influence the individual’s decision to use the tool. Perceptual behavior control is the individual’s perception of the relative difficulty of implementing a certain behavior [33]. This perception is affected by internal factors (such as skills, knowledge, and information acquisition) and external factors (such as time, money, and cost). In this study, subjective norms and perceptual behavior control influenced the intention of college students to use the tool. Therefore, the willingness to use the tool by residents can be increased by strengthening publicity, especially by inviting professionals or government initiatives to provide knowledge or money.

The present study has its limitations. First, the dietary nutrient density educational tool may be difficult to understand because it was developed through three examples which are not presented in the text. However, these three examples were given as a supplementary material. The other limitation of this study was that when the acceptance of use of nutrient-dense educational tools was investigated for residents, the teaching ability of the lecturer may affect the responses by residents to the questionnaire. However, we designate a person to give the lecture to reduce bias to a certain extent. Finally, the sample used in our analysis based the special population—college students. However, we plan to investigate among different groups of people and enlarge the sample size later.

## Conclusion

To our knowledge, we were the first to design a “dietary nutrient density educational tool” which extended the Chinese food guide pagoda (2022) helping residents understand the concept of nutrient density and making healthier food choices for improving their diet quality. Chinese college students from Henan province were investigated with their acceptance of using the “dietary nutrient density educational tool”. They were receptive to the concept of nutrient density and most of them would be willing to use the tool. Family residence, subjective norms and perceptual behavior control influenced their intention to use the tool. It suggested that the willingness to use the tool by residents can be increased by strengthening publicity, especially by inviting professionals or government initiatives or providing knowledge or money.

## Electronic supplementary material

Below is the link to the electronic supplementary material.

Supplementary Material 1

## Data Availability

No datasets were generated or analysed during the current study.
