# Supplementary material for: The development of a dietary nutrient density educational tool and the investigation of its acceptance by Chinese residents from Henan province
Source: BMC Public Health. 2024 Oct 4;24:2712. doi: 10.1186/s12889-024-20222-4 (PMC11452989; doi:10.1186/s12889-024-20222-4)
Supplement: Supplementary file 1 — Supplementary Material 1 [file 12889_2024_20222_MOESM1_ESM.docx]

**Supplementary material**

**Supplementary Table 1 Composition of recipes with higher NRF9.2 scores (in order of meal order)**

| **Meals times** | **Dish names** | **Food name and quantity** |
| --- | --- | --- |
| Breakfast | Mixed soybean milk | Soybean (dry) 15g, Red bean 12.5g |
|  | Quail eggs | Quail eggs 50g |
|  | Cold mixed bitter melon | Bitter melon 80g |
|  | Buckwheat steamed bun | Flour 55g, Tartary buckwheat flour 12.5g |
|  | Salt and oil | Salt 1g, Oil 4g |
| Lunch | Lentil steamed bun | Wheat flour 60g, Lentil flour 12.5g, Tartary buckwheat flour 12.5g |
|  | Seaweed salad | Kelp (soaked) 80g |
|  | Stir fried brassica campestris | Brassica campestris 80g |
|  | Stir fried meat with sweet pepper | Sweet pepper 50g, Pork（lean）50g |
|  | Salt and oil | Salt 2g, Oil 11g |
| Dinner | Highland barley steamed bun | Wheat flour 60g, Highland barley flour 25g |
|  | Stir fried water spinach | Water spinach 50g, Chili pepper 30g |
|  | Stir fried scallops with chili peppers | Chili peppers 30g, Leeks 60 g, Scallops 50g |
|  | Salt and oil | Salt 2g, Oil 10g |
| Snack | Yam | 75g |
|  | Milk | 300ml |
|  | Mango | 150g |
|  | Walnut | 10g |
|  | Strawberry | 150g |

**Supplementary Table 2 Composition of recipes with higher NRF9.2 scores (in order of food categories)**

| **Food category** | **block number** | **Food name** | **NRF9.2 Score** | **Amount to** |
| --- | --- | --- | --- | --- |
| First layer: Cereals (5 blocks) | 1^st^ block | Tartary buckwheat flour（0.5 block） | 3 | 4 points：4 blocks；  3 points：1 block； |
|  |  | Highland barley（0.5 block） | 4 |  |
|  | 2^nd^ block | Lentils, red beans（0.5 block） | 3 |  |
|  |  | Wheat flour（0.5 block） | 4 |  |
|  | 3^rd^ block | Wheat flour | 4 |  |
|  | 4th block | Wheat flour | 4 |  |
|  | 5th block | Wheat flour | 4 |  |
| Second layer: Vegetables (2 blocks) | 1^st^ block | Bitter gourd, spinach, and water spinach | 4 | 4 points：1.5 blocks  3 points：0.5 block |
|  | 2^nd^ block | Kelp, sweet pepper（0.5 block） | 4 |  |
|  |  | Pepper and leek（0.5 block） | 3 |  |
| Second layer: Fruits (2 blocks) | 1^st^ block | Mango | 4 | 4 points：2 blocks |
|  | 2^nd^ block | Strawberry | 4 |  |
| Third layer: Lean meat, eggs and fish(3 blocks) | 1^st^ block | Pork (lean) | 3 | 3 points：2 blocks  4 points：1 block |
|  | 2^nd^ block | Quail egg | 3 |  |
|  | 3^rd^ block | Pectinid | 4 |  |
| Fourth layer: Soy beans, nuts and milk and dairy products(2 blocks) | 1^st^ block | Soybean (dry)（0.5 block） | 3 | 4 points：1.5 blocks；  3 points：0.5 block |
|  |  | Walnut（0.5 block） | 4 |  |
|  | 2^nd^ block | Milk | 4 |  |
| Fifth layer: Oil and salt(1 block) | 1^st^ block | Peanut oil（0.5 block） | 3 | 3 points：0.5 block |
|  |  | Salt（0.5 block） | --- | Not participating in drawing |

**Supplementary Table 3 Composition of recipes with moderate NRF9.2 scores (in order of meal order)**

| Meals times | Dish names | Food name and quantity |
| --- | --- | --- |
| Breakfast | Bread | Bread 75g |
|  | Tofu (North) | Tofu (North) 70g |
|  | Stir fried cauliflower | Cauliflower 80g |
|  | Yogurt | Yogurt 150g |
|  | Duck eggs | Duck eggs 50g |
|  | Salt and oil | Salt 1g, Oil 4g |
| Lunch | Mung bean rice | Rice 75g, Mung bean 25g |
|  | Garlic sprouts | Garlic sprouts 50g |
|  | Lotus root | Lotus root 62g |
|  | Flatfish | Flatfish 50g |
|  | Beans | Beans 80g |
|  | Salt and oil | Salt 2g, Oil 11g |
| Dinner | Millet and pinto beans congee  Macaroni | Millet 25g, Pinto bean 25g  Macaroni 50g |
|  | Pork ( lean) | Pork ( lean) 50g |
|  | Asparagus lettuce | Asparagus lettuce 80g |
|  | Lettuce | Lettuce 100g |
|  | Salt and oil | Salt 2g, Oil 10g |
| Snack | Yogurt | 150g |
|  | Apple | 150g |
|  | Cashew | 10g |
|  | Persimmon | 150g |
|  | Sweet potato | 75g |

**Supplementary Table 4 Composition of recipes with moderate NRF9.2 scores (in order of food categories)**

| **Food category** | **Portion number** | **Food name** | **NRF9.2 Score** | **Amount to** |
| --- | --- | --- | --- | --- |
| First layer: Cereals (5 blocks) | 1^st^ block | Bread | 2 | 4 points：0.5 block；  3 points：1.5 blocks  2 points：3 blocks |
|  | 2^nd^ block | Rice | 2 |  |
|  | 3^rd^ block | Millet and Rice | 2 |  |
|  | 4th block | Mung bean（0.5 block） | 4 |  |
|  | 5th block | Pinto bean（0.5 block） | 3 |  |
|  |  | Macaroni | 3 |  |
| Second layer: Vegetables (2 blocks) | 1^st^ block | Lettuce and cauliflower | 3 | 3 points：1.5 blocks  1 points：0.5 block |
|  | 2^nd^ block | Asparagus lettuce（0.5 block） | 3 |  |
|  |  | Lotus root and garlic stem（0.5 block） | 1 |  |
| Second layer: Fruits (2 blocks) | 1^st^ block | Apple | 3 | 3 points：2 blocks |
|  | 2^nd^ block | Persimmon | 3 |  |
| Third layer: Lean meat, eggs and fish(3 blocks) | 1^st^ block | Lean pork | 3 | 3 points：2 blocks  2 points：1 block |
|  | 2^nd^ block | Flounder | 3 |  |
|  | 3^rd^ block | Duck's egg | 2 |  |
| Fourth layer: Soy beans, nuts and milk and dairy products(2 blocks) | 1^st^ block | Cashew（0.5 block） | 3 | 3 points：1.5 blocks  1 points：0.5 block |
|  |  | Tofu (North) (0.5 block) | 1 |  |
|  | 2^nd^ block | Yogurt | 3 |  |
| Fifth layer: Oil and salt(1 block) | 1^st^ block | Peanut oil（0.5 block） | 3 | 3 points：0.5 block |
|  |  | Salt（0.5 block） | --- | Not participating in drawing |

**Supplementary Table 5 Composition of recipes with lower NRF9.2 scores (in order of meal order)**

| **Meals times** | **Dish names** | **Food name and quantity** |
| --- | --- | --- |
| Breakfast | Salted duck egg | Salted duck egg 50g |
|  | Deep-fried dough sticks | Deep-fried dough sticks 90g |
|  | Soybean sprouts | Soybean sprouts 80g |
|  | Yogurt | Yogurt 200g |
|  | Salt and oil | Salt 2g, Oil 4g |
| Lunch | Rice | Rice 50g |
|  | Dried corn | Dried corn 25g |
|  | Lettuce leaves | Lettuce leaves 80g |
|  | Garlic sprouts | Garlic sprouts 112g |
|  | Fat pork | Fat pork 50g |
|  | Salt and oil | Salt 2.5g, Oil 11g |
| Dinner | Perch  Lotus Root | Perch 50g  Lotus Root 80g |
|  | Kidney beans | Kidney beans 25g |
|  | Deep-fried round and flat dough-cake | Deep-fried round and flat dough-cake 60g |
|  | Okra | Okra 38g |
|  | Onion | Onion 60 |
|  | Salt and oil | Salt 2.5g, Oil 10g |
| Snack | Yogurt | 100g |
|  | Banana | 150g |
|  | Grape | 150g |
|  | Soybean | 15g |
|  | Stir fried peanut kernels | 10g |
|  | Taro | 75g |

**Supplementary Table 6 Composition of recipes with lower NRF9.2 scores (in order of food categories)**

| **Food category** | **Portion number** | **Food name** | **NRF9.2 Score** | **Amount to** |
| --- | --- | --- | --- | --- |
| First layer: Cereals (5 blocks) | 1^st^ block | Kidney beans（0.5 block） | 2 | 2 points：1.5 blocks  1 points：3.5 blocks |
|  |  | Corn flour（0.5 block） | 1 |  |
|  | 2^nd^ block | Rice | 2 |  |
|  | 3^rd^ block | Deep-fried round and flat dough-cake | 1 |  |
|  | 4th block | Deep-fried dough sticks and Deep-fried round and flat dough-cake | 1 |  |
|  | 5th block | Deep-fried dough sticks | 1 |  |
| Second layer: Vegetables (2 blocks) | 1^st^ block | Soybean sprouts, lotus roots and onions | 1 | 2 points：0.5 block  1 points：1.5 blocks |
|  | 2^nd^ block | Garlic sprouts（0.5 block） | 1 |  |
|  |  | Lettuce leaves and Okra（0.5 block） | 2 |  |
| Second layer: Fruits (2 blocks) | 1^st^ block | Banana | 1 | 1 points：1 block  2 points：1 block |
|  | 2^nd^ block | Grape | 2 |  |
| Third layer: Lean meat, eggs and fish(3 blocks) | 1^st^ block | Fat pork | 1 | 1 points：2 blocks  2 points：1 block |
|  | 2^nd^ block | Perch | 2 |  |
|  | 3^rd^ block | Salted duck egg | 1 |  |
| Fourth layer: Soy beans, nuts and milk and dairy products(2 blocks) | 1^st^ block | Soybean（0.5 block） | 3 | 3 points：1.5 blocks；  1 points：0.5 block |
|  |  | Stir fried peanut kernels（0.5 block） | 1 |  |
|  | 2^nd^ block | Yogurt | 3 |  |
| Fifth layer oil and salt(1 block) | 1^st^ block | Peanut oil（0.5 block） | 3 | 3 points：0.5 block |
|  |  | Salt（0.5 block） | --- | Not participating in drawing |
